# Four Faces of Infective Endocarditis: Where Thinking Outside the Box Was Crucial

**DOI:** 10.3390/jcm14072162

**Published:** 2025-03-22

**Authors:** Ali El-Shamy, Patrick Man, Gayatri Vijapurkar, Baher Hanna, Attila Kardos

**Affiliations:** 1 Department of Cardiology, Translational Cardiovascular Research Group, Milton Keynes University Hospital NHS Foundation Trust, 8H Standing Way, Eaglestone, Milton Keynes MK6 5LD, UK; ali.elshamy@mkuh.nhs.uk (A.E.-S.); patrick.man@mkuh.nhs.uk (P.M.); gayatri.vijapurkar@mkuh.nhs.uk (G.V.); baher.hanna@mkuh.nhs.uk (B.H.); 2 Faculty of Medicine and Health Sciences, University of Buckingham, Buckingham MK18 1EG, UK

**Keywords:** infective endocarditis, endocarditis team, complex cases, CIED, PWID, relapsing ViV bMV endocarditis, subclinical I.E

## Abstract

**Background/Objectives:** Infective Endocarditis (IE) presents both a challenging diagnostic and treatment task. **Methods:** The incidence of IE has grown with the advancement in treatment technologies offered to patients, including intra-cardiac electrical device insertion and a variety of transcutaneous structural interventions. **Results:** Guidelines recommend the involvement of a multi-disciplinary approach to prompt diagnosis and decisions regarding treatment. **Conclusions:** We present the management conundrums of four complex cases of IE and highlight the importance of the Extended Endocarditis Team.

## 1. Introduction

Infective endocarditis (IE) remains a formidable clinical challenge. It affects 3–10 people per 100,000 annually, with a 30-day mortality rate of up to 30% [1]. The incidence of IE has risen due to advancements in cardiac care, including the increased use of intra-cardiac electronic devices (CIEDs) and interventions for structural heart disease. The International Cardiology Societies underscore the importance of a multidisciplinary approach for timely diagnosis and treatment decisions [2]. Effective IE management requires a delicate balance between eradicating the infection and addressing the patient’s unique circumstances. This often necessitates complex decisions to reconcile these competing priorities.

This article presents four cases of IE, and we discuss the pivotal role of integrated imaging modalities and specialised expertise in diagnosis and management (“thinking outside the box strategy”), emphasising the value and the role of the Extended Endocarditis Multidisciplinary Team (EEMT).

### 1.1. Case Series Presentation

#### 1.1.1. Case 1

***Background:***

A 82-year-old frail woman with a history of permanent pacemaker implantation for symptomatic bradycardia, thymoma resection for myasthenia gravis, and hypertension was admitted.

***Presentation:***

The patient presented to the emergency department with acute onset fever and malaise. Her examination was unremarkable, with no appreciated heart murmurs.

***Investigations:***

Initial investigations included a white cell count (WCC) of 8.3, and a C-Reactive Protein (CRP) of 196. Chest X-ray and urinalysis did not show signs of infection. Blood cultures (BCs) were taken at presentation.

***Clinical progress and treatment:***

**Figure 1:**
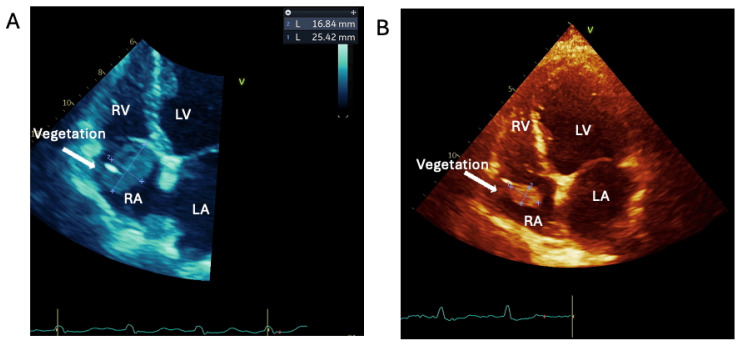
Apical 4-chamber view of the transthoracic echocardiography of case 1 with infected CIED in the right atrium with a large vegetation on the atrial site. (**A**): on admission, the size of the vegetation is 25 × 17 mm. (**B**): the vegetation 42 days after antibiotic IV and anticoagulation has reduced size to below 20 mm—this allowed the CIED to be safely extracted percutaneously. RV: right ventricle; LV: left ventricle; RA: right atrium; LA: left atrium.

Broad-spectrum antibiotics were started initially. These were adjusted to Flucloxacillin as her BC grew staphylococcus aureus, and the probability of IE was raised. Whilst her initial transthoracic echocardiogram (TTE) showed no vegetations or valve lesions, Rifampicin was added, given the strong suspicion of cardiac implantable electronic device (CIED)-related endocarditis. TTE was repeated one week later and showed a 25.4 mm × 16.8 mm right-atrial mass attached to the pacemaker lead, protruding through her tricuspid valve (TV); hence, CIED-related IE was confirmed (Figure 1A, Video S1A). The EEMT proposed the treatment options of percutaneous device extraction, surgical device removal, and thrombus debulking. Given the patient’s frailty and prior thoracic surgery, percutaneous extraction was selected as the preferred treatment strategy due to its lower surgical risk profile. It was also recommended to continue antibiotics and anticoagulation with Warfarin to reduce thrombus size to facilitate percutaneous extraction (aiming to reduce the size of the vegetation below 2 cm). Oral anticoagulant, i.e., Warfarin, was chosen to allow uninterrupted therapy during the procedure and minimise haematoma risk. After a further 3 weeks of antibiotics and anticoagulation treatment, the thrombus size reduced to 18 × 10 mm, and the pacemaker was safely extracted with no complications (Figure 1B, Videos S1B and S2A,B).

***Outcome and follow up***

Following a prolonged admission involving 10 weeks of Flucloxacillin and 4 weeks of rifampicin, the patient was safely discharged. Outpatients follow up BCs remained negative, and since the patient had no pacemaker reliance (first-degree atrioventricular block, normal axis deviation), she avoided a new pacemaker implantation. 

***Case 1—Timeline of events:***

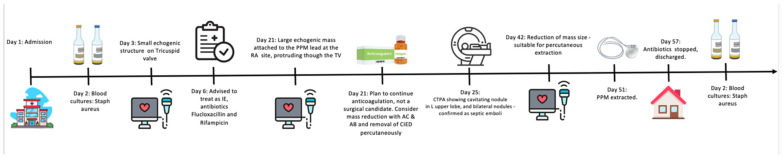

#### 1.1.2. Case 2

***Background:***

A 32-year-old lady with a background of being a person who injected drugs (PWID), a previous right-thigh abscess, right lower-limb deep vein thrombosis, and hepatitis B was admitted.

***Presentation:***

The patient was hospitalised with weakness, anorexia, bilateral flank pain, diarrhoea, intermittent vomiting, and a dry cough for one week. On examination, she was tachycardic with bilateral crackles, left-flank tenderness, and epigastric tenderness. No IE peripheral stigmata were noted.

***Investigations:***

Her initial blood tests showed a WCC of 14.7, d-dimer of 986, and CRP of 378, with normal renal function. Chest X-ray showed increased lung markings bilaterally, with predominant airspace consolidation at the right middle and lower lung.

***Clinical progress and treatment:***

**Figure 2:**
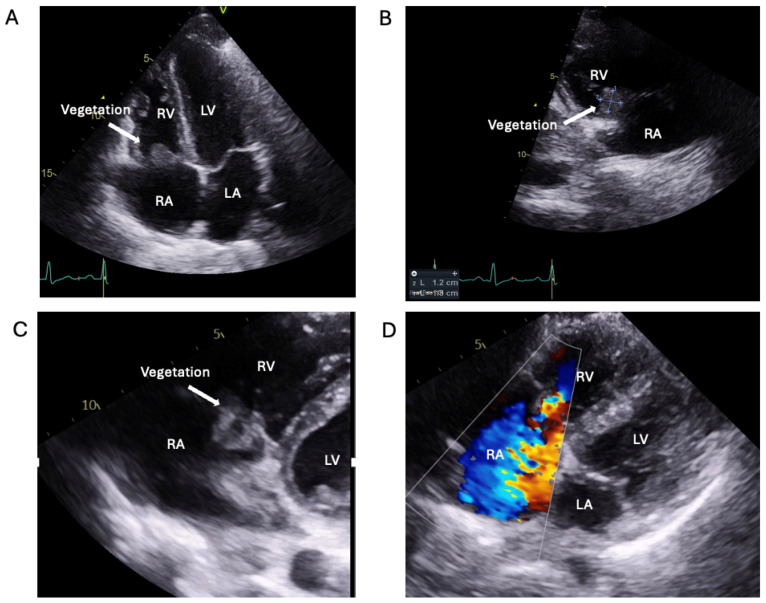
Transthoracic echocardiography of the right-sided IE of non-compliant high-risk young PWID with relapsing IE. (**A**): A4CV showing TV vegetation. (**B**): Modified parasternal long-axis view showing RV inflow and TV vegetation. (**C**): Modified parasternal long-axis view showing RV inflow and TV vegetation, after readmission post self-discharge. (**D**): Colour Doppler image of modified parasternal long-axis view showing RV inflow—Tricuspid Regurgitation (TR) after readmission. RV: right ventricle; LV: left ventricle; RA: right atrium; LA: left atrium.

After BCs were taken, broad-spectrum antibiotics were started empirically for suspected atypical community-acquired pneumonia. BCs subsequently grew staphylococcus aureus. Antibiotics were adjusted to Ceftriaxone and Metronidazole, and subsequently Flucloxacillin according to sensitivity. A TTE revealed vegetation on the septal (10 × 5 mm) and posterior (15 × 7 mm) leaflets of the TV, with moderate tricuspid regurgitation (Figure 2A,B, Video S3A,B). A whole-body CT scan identified a right-sided pulmonary embolus and widespread septic emboli. A Peripherally Inserted Central Catheter (PICC) line was inserted and the EEMT agreed to continue medical management with antibiotics in lieu of surgical treatment. With this, the patient’s clinical and biochemical status improved. The patient self-discharged against medical advice 14 days into the admission, prior to the completion of her antibiotic course. Six weeks later, the patient re-presented with infective symptoms. Flucloxacillin treatment was resumed after persistent IE was confirmed on repeat TTE and cultures (Figure 2C,D and Video S3C). This was temporarily switched to oral therapy as the patient lost intravenous access, until another PICC line was inserted. However, concerns were raised with regard to PICC line misuse. Intravenous antibiotic adherence was problematic as the patient again stated she would like to self-discharge. The EEMT recommended intravenous antibiotic Ortivancin with a >16 day half-life [3]. After administration, the PICC line was removed, and the patient was discharged home with oral Flucloxacillin to complete the six-week course.

***Outcome and follow up:***

The patient was scheduled for repeat BC, a further dose of Ortivancin, and cardiology follow up, but however did not attend any of these appointments. Two months later, she was seen in the venous thrombo-embolism clinic. She reported no further infection symptoms and was engaging in efforts to stop intravenous drug use. 

***Case 2—Timeline of events:***

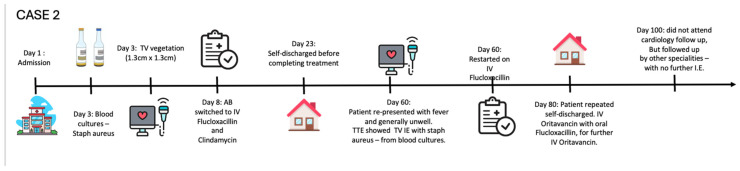

#### 1.1.3. Case 3

***Background:***

A 72-year-old lady with a 6-year history of bioprosthetic mitral valve replacement for obstructive hypertrophic cardiomyopathy with severe mitral regurgitation, dual-chamber pacemaker implantation, chronic obstructive pulmonary disease, and hypertension was admitted.

***Presentation:***

The patient presented with a sudden onset of severe central chest pain radiating to her left shoulder and back that woke her from sleep. She also reported progressive shortness of breath, difficulty climbing stairs, and unsteadiness. Examination revealed a diastolic murmur and a pansystolic murmur in the mitral valve area. Clear lung sounds were audible bilaterally with mild bilateral peripheral oedema extending to the knees.

***Investigations:***

Admission blood tests revealed a WCC of 9.6 and CRP of 116. Her admission chest X-ray showed no major collapse, consolidation, or oedema.

***Clinical progress and treatment:***

**Figure 3:**
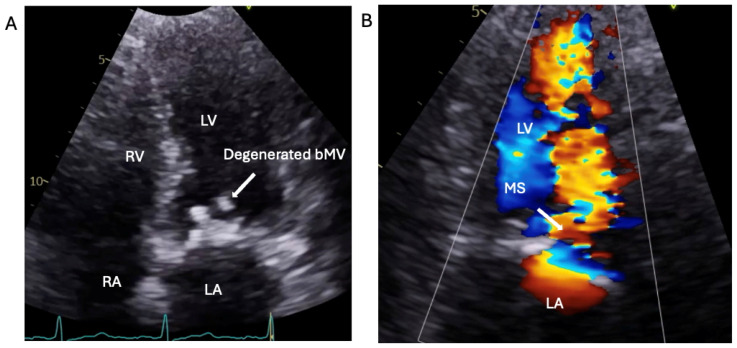
Apical 4-chamber view of transthoracic echocardiography of degenerated biological Mitral Valve (bMV), (white arrow). (**A**): A4CV showing calcified stenotic bMV. (**B**): Colour Doppler flow in diastole across degenerated bMV. RV: right ventricle; LV: left ventricle; RA: right atrium; LA: left atrium.

**Figure 4:**
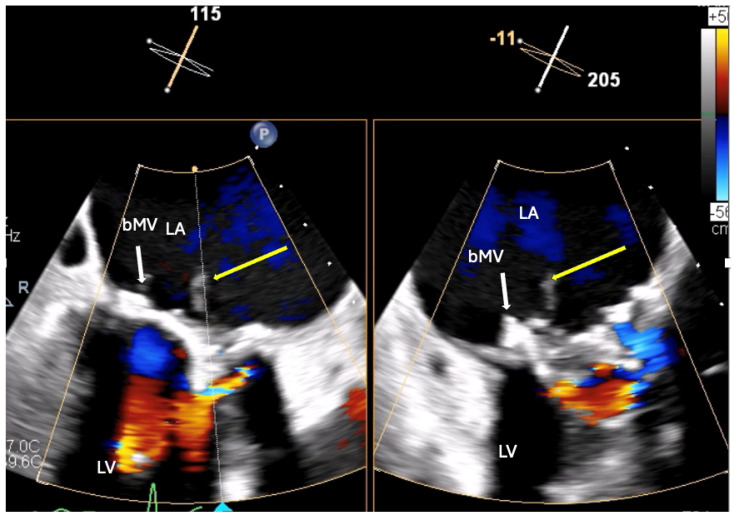
Transoesophageal echocardiography image—cross-sectional image of degenerated immobile mitral valve bioprosthesis (bMV) (yellow arrows indicate vegetation attached to mitral valve). LV: left ventricle; LA: left atrium.

**Figure 5:**
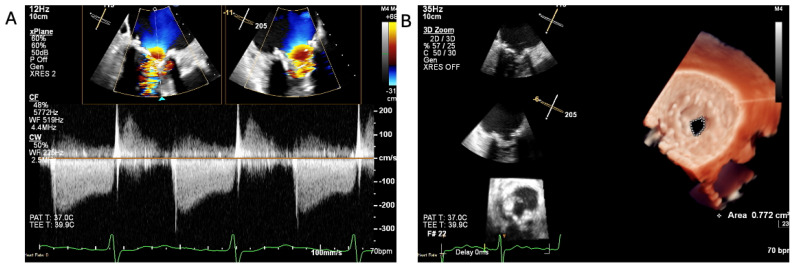
Transoesophageal echocardiography image of degenerated mitral valve bioprosthesis. (**A**): CW Doppler across stenotic mitral valve with mean gradient of 12 mmHg, (**B**): 3D planimetry mitral valve area (MVA); value of 0.77 cm^2^ in keeping with severe MS.

**Figure 6:**
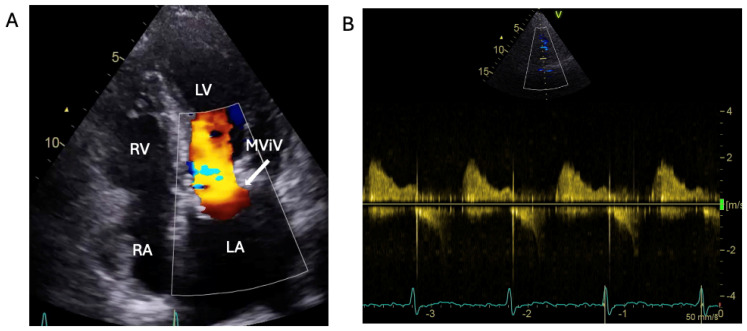
Transthoracic echocardiography colour Doppler and CW velocity after transcutaneous MViV intervention. (**A**): Colour Doppler flow of apical 4-chamber view of transthoracic echocardiography after percutaneous mitral valve-in-valve implantation (MViV). (**B**): Continuous Doppler velocity across MViV showing no significant inflow gradient. RV: right ventricle; LV: left ventricle; RA: right atrium; LA: left atrium.

The patient was treated with intravenous Co-Amoxiclav after BC revealed enterococcus faecalis. TTE and subsequent transoesophageal echocardiogram revealed restricted excursion of the tissue mitral valve suggestive of severe MS (MVA: 0.7 cm^2^) with a mobile, 1.4 cm long vegetation (Figure 3, Figure 4 and Figure 5, Video S4). The EEMT deemed it too high risk to redo valve replacement surgery. After completing a 6-week course of Teicoplanin with subsequent negative BCs, the patient electively underwent a mitral valve-in-valve replacement (MViV, Sapien balloon expendable) for severe mitral stenosis to improve the clinical signs of pulmonary congestion, with excellent clinical results (Figure 6, Video S5).

**Figure 7:**
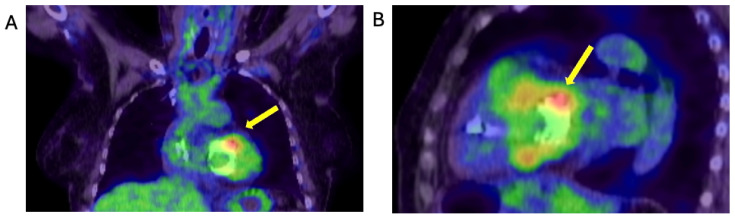
FDG-PET CT after MViV implantation and relapsing enterococcus faecalis bacteraemia. FDG-PET-CT images of chest show focal tracer accumulation over MVR (yellow arrows), which is also present on non-attenuation correction images, suggestive of underlying infective endocarditis. Cardiac pacemaker leads, and generator showed no tracer accumulation. (**A**): coronal and (**B**) sagittal PET CT images of chest.

A month after her intervention, the patient re-presented with presyncopal symptoms. Her BC revealed a relapse of the enterococcus faecalis IE. She completed a further Teicoplanin course through outpatient parenteral antibiotic therapy (OPAT). BCs during follow up were negative after completing antibiotics until after six-weeks of her follow up, when her repeat BCs grew enterococcus faecalis because of relapsing IE, confirmed by FDG-PET-CT with focal tracer accumulation over the MVR (Figure 7). The patient completed the subsequent six weeks of Amoxicillin and Gentamicin treatment. This was then stepped down to oral Amoxicillin and she was discharged home with ongoing antibiotics.

***Outcome and follow up:***

With careful microbiologist and cardiologist follow ups, she remained infection free until 2 weeks after stopping her antibiotics. For the 4th time, she relapsed with the same organism. At this point, the EEMT recommended *lifelong antibiotic* therapy since no further operative intervention was appropriate. She had no further relapsing IE on continued antibiotic treatment

***Case 3—Timeline of events:***

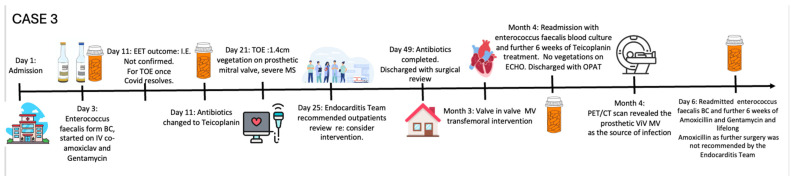

#### 1.1.4. Case 4

***Background:***

**Figure 8:**
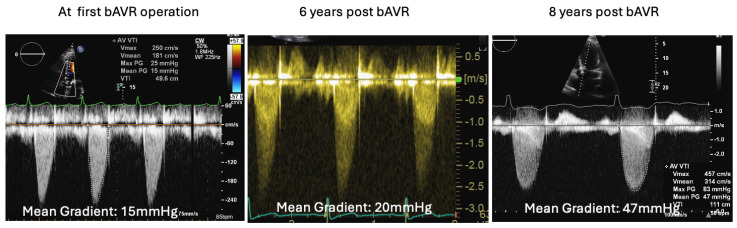
Series of transthoracic echocardiographies in valve clinic—CW measurements of transvalvular gradient across AV bioprosthesis since its implantation. Mean transvalvular gradient showed progressive increase suggestive of severe degenerative AS.

A 55-year-old gentleman with prior tissue aortic valve replacement (tAVR) for aortic root dilatation and severe aortic regurgitation 9 years prior—who had also been treated for hypertension and type 2 diabetes mellitus—was admitted. He was intermittently followed up in the valve clinic and was found to have a mild aortic stenosis of the tAVR (mean pressure gradient 15 mmHg), and 6 years later surveillance showed a slightly progressive moderate aortic stenosis (mean gradient: 20 mmHg) (Figure 8).

***Presentation:***

At outpatient review, he reported experiencing exertional dyspnoe and fatigability, and occasional episodes of chest tightness. Physical examination revealed a harsh ejection systolic murmur in the precordium.

***Investigations:***

**Figure 9:**
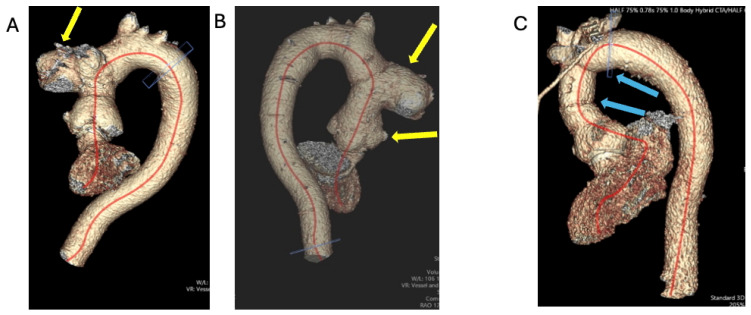
A three-dimensional rendering of CT aorta to show (**A**,**B**) the saccular pseudoaneurysm of the ascending aorta and dilated sinus of Valsalva (preoperatively, yellow arrows); (**C**) the replaced ascending aorta and proximal arch (post-operatively blue arrows).

TTE showed an ejection fraction >55% and moderate concentric left-ventricular hypertrophy, mild aortic root enlargement (4.1 cm), and a calcified severely stenosed tAVR with a mean gradient of 47 mmHg (Figure 8). CT coronary angiogram revealed normal coronaries, a well-seated calcified tissue aortic valve, and generalised dilation of the ascending aorta and aortic arch, suggestive of a large saccular pseudoaneurysm (42 mm by 50 mm) (Figure 9A,B).

***Clinical progress and treatment:***

**Figure 10:**
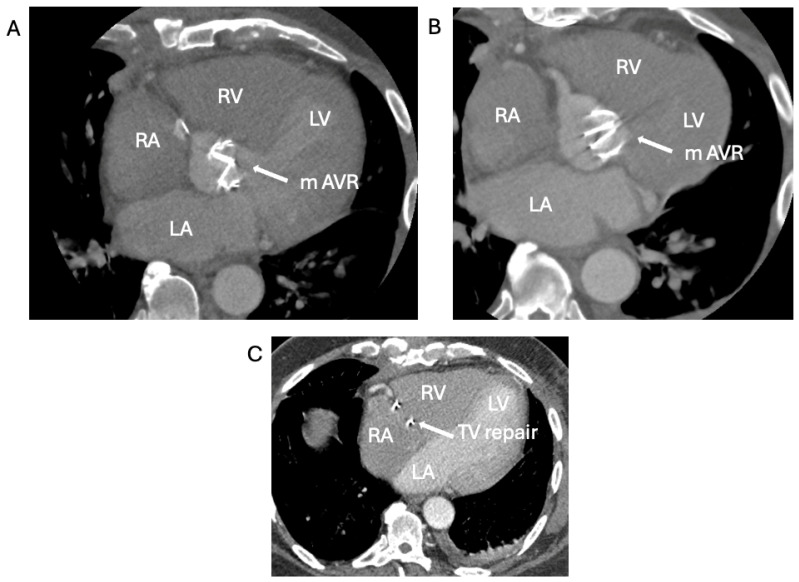
CT images of mechanical AV replacement and TV annuloplasty ring. (**A**): mechanical aortic valve in diastole. (**B**): mechanical aortic valve in systole. (**C**): TV annuloplasty ring.

**Figure 11:**
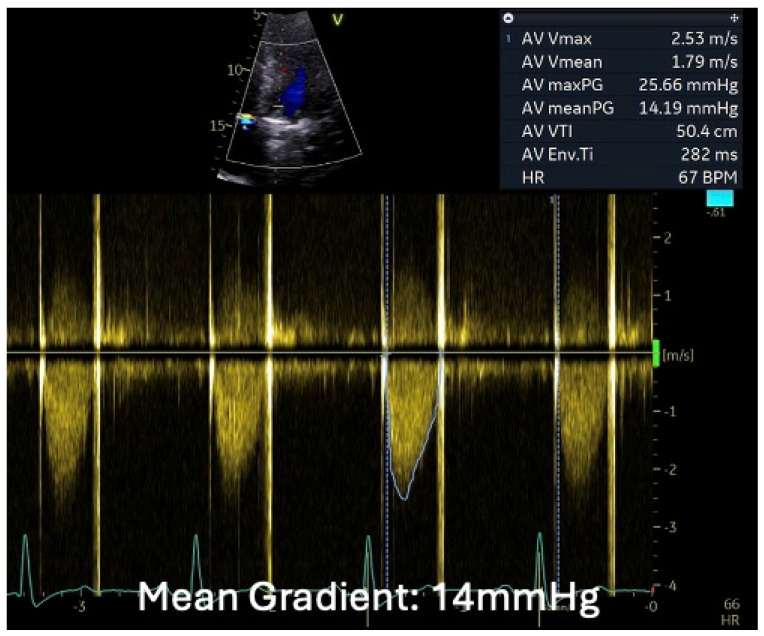
Transthoracic echocardiography—CW measurements of the transvalvular gradient across the mechanical AVR. After redoing the mechanical AVR and ascending Aorta replacement, the flow velocity across the mechanical AVR was normal.

The patient was referred to have their metallic aortic valve, aortic root, ascending aorta, and proximal arch replacement urgently replaced. A TV repair was also performed (Figure 9C, Figure 10A,B,C and Figure 11). The operation was prolonged and complicated by the rupture of a pseudoaneurysm, difficult aortic valve access, weaning, and hemostasis, respectively. IE was proposed as the underlying cause of the patient’s aortic valve degeneration when the tissue culture of the removed aortic valve and root grew coagulase-negative staphylococcus capitis and cutibacterium acne. During his post-operative intensive care stay, the EEMT advised Daptomycin and Rifampicin, with advice to complete a 6-week course according to sensitivity. His infection remained under control and clinically stable. On the day of his scheduled discharge, the patient developed a fever, and a subsequent BC revealed candida albicans. He was restarted on Caspofungin, which was switched to Fluconazole after ophthalmology review suggested possible candida albicans chorioretinitis. The patient developed intracranial hematoma as a consequence of the hemorrhagic transformation of his embolic stroke due to elevated INR caused by the interaction of the antifungal treatment and Warfarin. Following two weeks of stroke rehabilitation, the patient recovered well and was discharged home to complete a six-week course of antifungal treatment via OPAT.

***Outcome and follow up:***

Over the subsequent two months following discharge, the patient reported feeling well, made progress with his rehabilitation, and continued to have negative BCs. 

***Case 4—Timeline of events:***

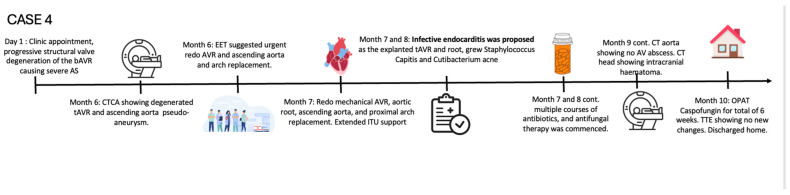

## 2. Discussion

EEMT played a key role in each of these cases, and this series highlights challenges in managing complex cases with IE not necessarily supported by the established guidelines.

### 2.1. The Conversion of High Risk Open-Heart-Surgery to Lower-Risk Lead-Extraction in a CIED-IE Case

**Case 1** presents a frail, multi-morbid lady with CIED-IE. The observed infection rate requiring device/lead replacement/extraction is between 1.5% and 6% [4,5,6], with a mortality rate of 5.5% and 14.6% by 30 day and 1 year, respectively [7]. Complete device extraction is advised in such cases, and given the size of the vegetation (thrombotic/infective material > 2cm), surgical removal is recommended. However, due to the patient’s comorbidities this was deemed too high risk. The EEMT instead decided to delay extraction, with usage of anticoagulation in addition to prolonged antibiotic therapy to reduce to a safe vegetation size suitable for percutaneous extraction. Anticoagulation is not usually indicated in patients with IE to avoid bleeding complications, especially in left-sided infections during septic cerebral embolisation. Admittedly, the evidence is absent, and it is recommended in such cases to discuss within the EEMT. Finally, the patient underwent a much-lower-risk procedure of percutaneous device extraction with full recovery without the need for pacemaker re-implantation.

### 2.2. Logistical Management Challenges in a Non-Compliant High-Risk Young PWID with Relapsing Right-Sided IE

Right-sided infective endocarditis (RSIE) is generally more benign compared with left-sided endocarditis, with lower mortality [8]. While guidelines indicate that most RSIE cases can be successfully treated with targeted antibiotic therapy, **Case 2** presented a complex scenario of non-adherence and premature treatment discontinuation. This necessitated the EEMT to revise the management strategy. The patient had TV vegetation and methicillin-sensitive staphylococcus aureus bacteraemia, typically treated with 4–6 weeks of Flucloxacillin. Guidelines also suggest RSIE affecting the TV to be considered for surgery [2]. However, treatment with antibiotics was selected in the first instance due to her complex history. It was not possible to limit treatment to a 2-week course due to septic pulmonary emboli. To ensure adequate antimicrobial exposure, the EEMT explored alternative treatment options with Oritavancin, a lipoglycopeptide antibiotic with a prolonged half-life of 393 h [9]. This allowed for a simplified dosing regimen, making it a viable option for patients with adherence challenges. This is an evidence-free area of management, but the local EEMT recommendation was the best possible choice for the best possible outcome. The guidelines suggest RSIE affecting the TV to be considered for valve surgery [2]. However, due to her social history and despite multiple discussions with the patient and various professionals, including drug misuse experts, a non-operative solution was agreed upon. An AngioVac suction thrombectomy device for percutaneous vegetation removal has been used for right-sided valvular endocarditis [10].

### 2.3. The Saga of Relapsing IE of the Prosthetic Mitral Valve Causing Structural Valve Degeneration and Transcutaneous MViV Prosthesis

Enterococci accounts for ~10% of valvular endocarditis cases, of which enterococcus faecalis is the major contributing organism. Its ability to colonise epithelial tissues and prosthesis, and subsequently form biofilms, makes it difficult to treat [11]. As such, it frequently presents with relapses [12]. **Case 3** highlights the management challenges posed by enterococci, and the role of the EEMT in finding the optimal solution. In cases of prosthetic valve endocarditis, guidelines advise meticulous debridement of the infected material. However, this was deemed too high risk for this patient, and the EEMT agreed on MViV intervention, accepting the high risk of re-infection [13,14]. The procedure allowed for the patient to have excellent symptomatic recovery yet later the patient suffered from relapses from enterococcus IE. The EEMT recommendation was to continue with *lifelong antibiotics* at further relapse, despite the guideline recommendation to remove the infected valve in such a situation. Further surgery would have carried unacceptable risk.

### 2.4. When the Diagnosis of IE Is Only Confirmed by the Tissue Culture of the Removed Degenerated tAVR

Polymicrobial IE is usually much rarer compared to monomicrobial IE, with an average range of 5.9% compared to 81.7% [15]. The sensitivity of the different organisms’ in polymicrobial endocarditis can pose a challenge in selecting the right antimicrobials. IE secondary to cutibacterium acnes is often difficult to diagnose as it does not usually present with fever or signs of inflammatory response. Valve dysfunction and the need for cardiac surgery have been reported [16,17]. In **Case 4**, the insidious onset of symptoms and the progressive, albeit slow, development of valvular stenosis of the tAVR were predominantly assessed based on clinical symptoms. IE was proposed as an underlying inflammatory etiology of the patient’s tAVR degeneration, based on the removed tissue culture that grew cutibacterium acnes and staphylococcus capitis. This case is an example of subclinical IE that can cause degenerative disease, especially in an implanted valve. Case 3 and 4 may share the same inflammatory pathophysiology of degenerative biological valve stenosis in the mitral and aortic position. A recent review comprehensively summarised the atypical features of IE to improve diagnostic accuracy in an at-risk population [18].

## 3. Conclusions

These four cases underscore the complexity of IE and the vital role of the EEMT in their management. While ESC guidelines offer a valuable framework, the guideline-directed medical therapy of IE is based on observational studies and expert opinion. The need and the utility of the EEMT, in complex cases with the advice and intuition of individual professional stakeholders, is well documented. We hope our case series can provide useful examples and discussion points for professionals caring for such patients.

### Learning Objectives

1. To recognise the value of the EEMT in making expert recommendations for frail patients with CIED endocarditis not suitable for open heart surgery.

2. To value the role of the EEMT recommendation in managing PWID without long-term compliance and a high risk of PICC line misuse.

3. To recognise the role of the EEMT in managing relapsing IE in a transcutaneous MViV prosthesis.

4. To recognise subclinical valvular infection as the pathophysiology of the degeneration of bioprostheses.

## Data Availability

The data supporting the findings of this case report are available in this published article. Further details are available from the corresponding author on reasonable request.

## Supplementary Materials

The following supporting information can be downloaded at https://www.mdpi.com/article/10.3390/jcm14072162/s1, Table S1: Summary of the cases of I.E.; Videos S1–S4 captures.

## List of Abbreviations and Acronyms

EEMT	Extended Endocarditis Multidisciplinary Team
CIED	Cardiac Implantable Electronic Device
PWID	Person Who Injected Drugs
ViV	Valve in Valve
IE	Infective Endocarditis
WCC	Wight Cell Count
CRP	C-Reactive Protein
PICC	Peripherally Inserted Central Catheter
OPAT	Outpatient Parenteral Antibiotic Therapy
TTE	Trans Thoracic Echocardiogram
ASA score	American Anaesthetists Society score
TV	Tricuspid Valve
ESC	European Society of Cardiology
tAVR	tissue Aortic Valve Replacement
RSISE	Right-Sided Infective Endocarditis
